# Controlling Fluid Diffusion and Release through Mixed-Molecular-Weight Poly(ethylene) Glycol Diacrylate (PEGDA) Hydrogels

**DOI:** 10.3390/ma12203381

**Published:** 2019-10-16

**Authors:** Kieran O’Donnell, Adrian Boyd, Brian J. Meenan

**Affiliations:** Nanotechnology and Integrated BioEngineering Centre (NIBEC), School of Engineering, Ulster University, Shore Road, Newtownabbey BT37 0QB, UK; ODonnell-k9@ulster.ac.uk (K.O.); ar.boyd@ulster.ac.uk (A.B.)

**Keywords:** poly(ethylene) glycol diacrylate, PEGDA, mesh size, diffusion coefficient, partition coefficient

## Abstract

Due to their inherent ability to swell in the presence of aqueous solutions, hydrogels offer a means for the delivery of therapeutic agents in a range of applications. In the context of designing functional tissue-engineering scaffolds, their role in providing for the diffusion of nutrients to cells is of specific interest. In particular, the facility to provide such nutrients over a prolonged period within the core of a 3D scaffold is a critical consideration for the prevention of cell death and associated tissue-scaffold failure. The work reported here seeks to address this issue via fabrication of hybrid 3D scaffolds with a component fabricated from mixed-molecular-weight hydrogel formulations capable of storing and releasing nutrient solutions over a predetermined time period. To this end, poly(ethylene) glycol diacrylate hydrogel blends comprising mixtures of PEGDA-575 Mw and PEGDA-2000 Mw were prepared via UV polymerization. The effects of addition of the higher-molecular-weight component and the associated photoinitiator concentration on mesh size and corresponding fluid permeability have been investigated by diffusion and release measurements using a Theophylline as an aqueous nutrient model solution. Fluid permeability across the hydrogel films has also been determined using a Rhodamine B solution and associated fluorescence measurements. The results indicate that addition of PEGDA-2000 Mw to PEGDA-575 Mw coupled with the use of a specific photoinitiator concentration provides a means to change mesh size in a hydrogel network while still retaining an overall microporous material structure. The range of mesh sizes created and their distribution in a 3D construct provides for the conditions required for a more prolonged nutrient release profile for tissue-engineering applications.

## 1. Introduction

Poly(ethylene) glycol (PEG)-based hydrogels have been investigated extensively for a range of biomedical applications due to their excellent biocompatibility, non-immunogenicity and resistance to protein adhesion [1,2,3,4]. The main areas of application include devices for wound healing, tissue-engineering constructs and drug-delivery systems within the pharmaceutical industry [5,6]. Hydrogels are networks of crosslinked polymer chains which are capable of absorbing and retaining water and soluble molecules therein. PEG itself forms polymer networks with relatively limited properties. However, the addition of key functional groups, such as acrylates to form PEG-methacrylate (PEGMA) and PEG-diacrylate (PEGDA), allows the polymer to be crosslinked via physical or chemical means, producing hydrogels with a range of properties [7,8,9,10,11]. In addition, ethylene glycol can be used to crosslink other polymers, such as in the case of PMMA crosslinked via EGDMA at various concentrations through the process of syneresis. This process results in phase separation during that polymerization, that causes the formation of micro-spherical particles which join together to form the porous network [12,13].

Typically, PEG-acrylate-based hydrogels are made from high-molecular-weight forms of the materials, in the range 2000–20,000, which when cross-linked, provide a relatively large mesh size that is capable of storing nutrients, drugs or other bioactive solutes [14,15]. For applications that require a hydrogel to absorb a biological payload and to then allow for its subsequent release, control of the mesh size is a major consideration. If the mesh dimensions are very large, diffusion can occur too readily, making it difficult to control both the amount of active payload within the hydrogel and its subsequent release. Likewise, if the mesh is too small, fluid diffusion is restricted, and the hydrogel cannot provide the required dose-related function [16,17,18].

This paper presents data for a range of crosslinked, mixed-molecular-weight PEGDA-based hydrogel networks that have been prepared via UV polymerization with the aim to control fluid flow within the resultant polymer structures by varying the mesh size. The core requirements to be met are biocompatible systems suitable for the storage of soluble bioagents at known concentrations and control of their subsequent release over time at doses relevant to nutrient needs of cells in tissue-engineering scaffolds. As the hydrogels of interest here will be a component in a hybrid scaffold system, their inherent mechanical properties are not a significant consideration other than their having robustness to handling in the context of an additive manufacturing process.

The loading and release of fluid by the hydrogel networks of interest have been investigated using a Theophylline (1,3-dimethylxanthine) solution as a model nutrient system [19,20,21]. Measurements have been made for both the diffusion and subsequent release of known concentrations of Theophylline. The nature of fluid permeability across the hydrogel films has been further considered via Rhodamine B diffusion and associated fluorescence measurements. The effect of the photoinitiator concentration on the provision of the required mesh dimensions has also been investigated.

## 2. Materials and Methods

### 2.1. Hydrogel Preparation

Poly(ethylene) glycol diacrylate (PEGDA) (Sigma Aldrich, Irvine, UK) of molecular weight 575 Da (PEGDA575) and 2000 Da (PEGDA2000) were individually mixed in distilled water to produce pre-polymerisation stock solutions at weight/volume concentrations of 20% and 40% in each case. These individual PEGDA575-2000 solutions were then blended together to obtain ratios of 100/0, 90/10, 80/20 and 70/30 PEGDA575/PEGDA2000, to give both 20% and 40% solutions of each blend. The photoinitiator 2-hydroxy-4′-(2-hydroxyethoxy)-2-methylpriophenone (Irgacure 2959, Sigma Aldrich, Irvine, UK) was dissolved in each of the polymer blend solutions at weight/volume concentrations 0.05% and 0.1%. It was found that ratios of PEGDA575-2000 of 50:50 ratio above do not fully polymerize in a time frame that is suitable for the fabrication of the hybrid tissue-engineering scaffolds concerned. To this end, the cure time required for the 70:30 system meant that this was deemed to be the highest ratio for these studies.

A 400 µL aliquot of each solution was dispensed between two clean glass slides, separated by a spacer of either 0.3 mm or 0.6 mm thickness and polymerized under UV light at 365 nm to create a cured hydrogel sheet. Sets of individual discs of 8 mm and 10 mm diameter were cut from each of the polymerized hydrogel sheets and placed in distilled water for 24 h at room temperature to fully swell the matrix and remove any excess unreacted soluble unreacted polymer and/or photoinitiator. Whereas, it is expected that 3D hydrogel scaffolds would be exposed to body temperatures, i.e., ~37 °C, the overall mesh size will not increase substantially from that measured at room temperature for the relatively low-molecular-weight systems of interest here.

### 2.2. Swelling Measurements of PEGDA575 and PEGDA575-2000 Hydrogels

Hydrogel discs were removed from distilled water and the surfaces carefully dried with blotting paper before being weighed to give a value for the wet polymer mass, W_w_. The samples were then dried in an oven at 40 °C overnight and reweighed to give the dry polymer mass, W_d_. A value for the swelling capacity, Q, for each of the hydrogels was calculated as the ratio of W_w_/W_d_. Mesh size calculations were conducted for the 100-0 PEGDA575-2000 system to obtain the average distance between crosslinks and the associated PEGDA575 mesh size. Statistical analysis was conducted using a one-way ANOVA.

### 2.3. Theophylline Entrapment and Release from PEGDA575-2000 Hydrogels

The free-volume theory for diffusion of a solute (carried in a fluid) through a permeable hydrogel membrane is dependent on the swelling characteristics and the solute size, according to the relationship [22] represented in Equation (1):

(1) $$\ln\left(\frac{D_{m}}{D_{0}}\right)=\Phi_{2}\left(\frac{-Bq_{2}}{V_{f}}\right)\left(\frac{1}{H}-1\right)$$

where *D_m_* and *D*_0_ are the diffusion coefficients of the solute in the hydrogel and in water, respectively, H is the volume fraction of water, *V_f_* is the free volume of the hydrogel, *B* is a constant, *q*_2_ is the cross-sectional area of the solute and Φ_2_ is the screening effect of the polymer network.

The relationship between and 1/*H* and ln(*D_m_*/*D*_0_) for the hydrogel mesh networks of interest here have been investigated using values of *D_m_* calculated for a Theophylline solution as a model nutrient solute system. Measurements have been made for both diffusion and the subsequent release of known concentrations of Theophylline. 

Theophylline (1,3-dimethylxanthine, Sigma Aldrich, Irvine, UK) was dissolved in distilled water at weight/volume concentration of 5 mg/mL and used to prepare the pre-polymerization hydrogel solutions, as described in Section 2.1. The polymerized discs prepared in this way were gently dried with blotting paper, and weighted to give the Theophylline wet polymer mass, W_w_. The Theophylline-loaded discs were rinsed briefly (10 s) in distilled water to remove any surface-bound residual material and placed in 10 mL of a 0.8% saline solution. The solution was removed and replaced at time points 30, 60, 180, 360, 540, 720 and 1440 min. UV-Vis measurements (Lambda 35, Perkin–Elmer, Beaconsfield, UK) were used to determine the Theophylline content of the solution released from the hydrogel discs into saline during the respective time periods. A simple Beer–Lambert Law calculation, employing a suitable stock solution dilution calibration curve, was used to quantify the Theophylline concentration in each solution [23].

### 2.4. Permeability of Rhodamine B through PEGDA575-2000 Hydrogels

The nature of fluid permeability across the hydrogel films has been further considered via Rhodamine B diffusion and associated fluorescence measurements [20,24,25]. The effect of the photoinitiator concentration on the provision of the required mesh dimensions has also been investigated.

**Figure 1:**
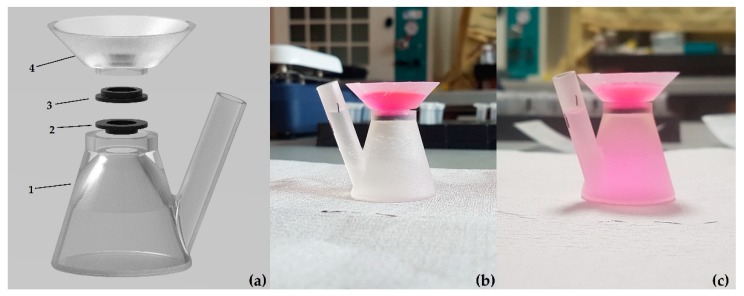
(**a**) Component parts of vertical transwell system, 3D-printed using Formlabs clear (1,4) and flexible (2,3) materials, wherein a Mixed-Molecular-Weight Poly(ethylene) Glycol Diacrylate (PEGDA) disc is placed between parts 2 and 3. Friction fit between parts 1 and 4 ensures that the system remains together; (**b**) transwell system at t = 0 h; (**c**) transwell system at t = 12 h.

Solute permeability studies through the various hydrogel membranes were conducted using a bespoke vertical transwell system, as shown in Figure 1, consisting of a 13.3 mL capacity top chamber fabricated from clear resin in a 3D SLA printer (Formlabs, Berlin, Germany), a middle open well area, again made of Formlabs flexible resin into which 8 mm² hydrogel samples are placed between 2 gaskets seated above and below the well and a bottom fluid collection chamber fabricated in a similar manner to the top component.

A solution of 0.1% weight/volume Rhodamine B in distilled water was prepared and 200 µL aliquots aspirated into the top chamber of the vertical transwell device, which was pre-filled with distilled water to maintain constant volume and hydrostatic pressure. The membranes tested ranged from 0.3 to 0.8 mm in thickness. The Rhodamine B content retained within the hydrogel samples was measured via fluorescence spectroscopy using a GENios FL plate reader (Tecan AG, Männedorf, UK) operating at 530 nm and 632 nm for excitation and emission wavelength settings, respectively.

The membrane permeability coefficient was calculated using Equation (2):

(2) $$P_{m}=\;-\left(\frac{Vh}{2At}\right)\ln\left(1-\frac{2C_{t}}{C_{0}}\right)$$

where *V* is cell volume, h and A are membrane thickness and exposed membrane area, respectively, and *C*_0_ and *C_t_* are original solute concentration (in the top chamber) and the collection chamber concentration at time *t*, respectively.

### 2.5. Partition and Diffusion Coefficient

For each hydrogel formulation, 10 pre-swollen discs, ranging in thickness from 0.3 to 0.8 mm were submerged in 3 mL of a 0.01% weight/volume Rhodamine B distilled water solution, representing *C*_0_ in Equation (2). After 48 h, the discs were removed from the solution and the Rhodamine B concentration, *C_s_*, measured via UV-Vis spectroscopy (Lambda 35, Perkin-Elmer, UK) at a wavelength of 270 nm. The partition coefficient, *K_d_*, was then calculated from these data using Equation (3):

(3) $$K_{d}=\frac{V_{s}\left(C_{0}-C_{s}\right)}{V_{m}C_{s}}$$

where *V_s_* and *V_m_* are the chamber cell volume and hydrogel volume, respectively.

The diffusion coefficient, *D_m_*, was calculated from Equation (4), using values for *P_m_* as calculated from Equation (2):

(4) $$D_{m}=\frac{P_{m}}{K_{d}}$$

### 2.6. Mesh Size Calculations

Mesh calculations were made for hydrogel discs created from 100% PEGDA575 (100-0) using Equation (5), where *Q* is the swelling ratio, *ρ* is equal to the density of PEGDA575 and *ρ*H_2_O the density of the solvent, i.e., deionized water.

(5) $$V_{2s}=\;\frac{1}{Q\frac{\rho }{\rho H_{2}0}+1}$$

From these data, the average molecular weight between crosslinks, 1/*M_c_*, can be determined from the Peppas–Merril model [26], using Equation (6), where *M_n_* is the average molecular weight of the polymer, *V*_1_ is the molar volume of water and χ is the Flory–Higgins polymer–solvent interaction parameter (0.426) [27].

(6) $$\frac{1}{M_{c}}=\;\frac{2}{M_{n}}-\;\frac{\frac{1}{V_{1}}[\ln\left(1-V_{2s}\right)+V_{2s}+\chi V_{2s}^{2}}{\rho \;\left[{\left(\frac{V_{2s}}{V_{2r}}\right)}^{\frac{1}{3\;}}-\;\frac{1}{2}\left(\frac{V_{2s}}{V_{2r}}\right)\right]}$$

Determination of the average end-to-end distance of the polymer chains can subsequently be calculated from Equation (7), where *l* is the bond length along the polymer backbone, which is 1.54 Å for PEG [28], *C_n_* is the Flory characteristic ratio, and *M_r_*, the repeating units molecular weight.

(7) $${\left(r_{0}^{-2}\right)}^{\frac{1}{2}}=l\left(\frac{2C_{n}M_{c}}{M_{r}}\right)$$

Equations (8) and (9) can be used to determine the extension ratio, *a*, allowing for the final calculation of mesh size, ξ.

(8) $$\alpha =v_{2s}^{-\frac{1}{3}}$$

(9) $$\xi =\;\alpha {\left(r_{0}^{-2}\right)}^{\frac{1}{2}}$$

While Equations (5)–(9) are suitable for calculating mesh size in polymer networks involving a single molecular weight, they do not consider the effects of mixed-molecular-weight contributions, as is the case here. Hence, to estimate the mesh size of the hydrogels created from the various PEGDA575-2000 formulations, Equation (10), as proposed by Ratner and Miller, [29] can be used for systems of pore radius ranging from 10–1000 Å, where *r_s_* is the solute radius, *r_p_* is the pore radius, *D_m_* is the diffusion coefficient of the material and *D*_0_ is the diffusion coefficient of the solute.

(10) $$\frac{D_{m}}{D_{0}}=\left[1-{\left(\frac{r_{s}}{r_{p}}\right)}^{2}\right].[1-2.104\left(\frac{r_{s}}{r_{p}}\right)+2.09{\left(\frac{r_{s}}{r_{p}}\right)}^{3}-0.95{\left(\frac{r_{s}}{r_{p}}\right)}^{5}$$

### 2.7. BET Surface Area Analysis

The Brunauer–Emmett–Teller (BET) surface area analysis was used to characterise the physical properties of the PEG575-2000 hydrogels created by crosslinking 20% and 40% polymers solutions with the 0.05% photoinitiator concentration. Adsorption and desorption BET isotherms were recorded at 77 K in a relative pressure range of 0.05 to 0.5 for freeze-dried samples (24 h) of each hydrogel system utilizing a Quantachrome Autosorb-1 instrument (Quantachrome, Hartley Wintney, UK). Specimens were degassed at 30 °C for 2 h prior to the analysis. Non-Local Density Functional Theory (NLDFT) using a slit pore model was applied to the surface area isotherm data for determination of pore radius for what are assumed to be nanopores of different geometries.

## 3. Results

### 3.1. Swelling of PEGDA 575 and PEGDA 575-2000 Hydrogels

**Figure 2:**
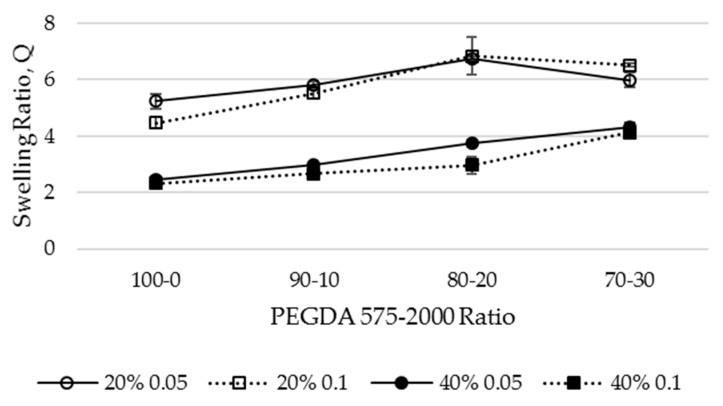
Difference in swelling ratio (Q) for various PEGDA575-2000 formulations.

Measurements of swelling ratio, Q, are provided in Figure 2 and indicate that there are differences between the hydrogel matrices created from the 20% and 40% PEGDA575 (100-0) formulations at both the 0.05 and 0.1 w/v% photoinitiator concentrations. This is to be expected, as at a higher polymer concentration, the distance between crosslinks within the polymer mesh decreases and the corresponding mesh size increases, as confirmed by the data presented in Table 1. Moreover, there is a statistically significant difference (*p* < 0.05) (n = 5) between the mesh sizes created with the 0.05% and 0.1% photoinitiator for both the 20% and 40% polymer concentrations.

A statistically significant difference (*p* < 0.05) is also observed for the swelling ratio of discs created with increasing PEGDA2000 additions to PEGDA575 at both the 20% and 40% polymer concentrations created with the 0.05% photoinitiator. However, for the discs prepared with the 0.1% photoinitiator concentration, statistically significant differences (*p* < 0.05) are only observed for the PEGDA575-2000 90-10 ratio for both the 20% and 40% solutions and not between the 80-20 and 70-30 at 20% or the 90-10 and 80-20 at 40%.

Whereas the data reported in Figure 2, indicates that the 70-30 formulation polymerizes within this (~30 min) time period at 20% polymer concentration, the Q value has decreased compared to that measured for the 80–20 hydrogel. However, this is still greater than that for the 100–0 system. This suggests that at both photoinitiator concentrations, the cross-linking efficiency for this 20% 70–30 solution is reduced by the presence of more of the PEGDA2000 polymer chains. By comparison, the trend in the 40% polymer solutions increases with PEGDA2000 contribution but with Q values less than those for the 20% case. This is interpreted as a limitation in the amount of PEGDA2000 loading that can increase the swelling ratio for the 20% polymer solution concentration.

### 3.2. Theophylline Loading and Release

To determine the amount of Theophylline solution that can be stored within the hydrogel discs, the content after polymerisation but before swelling was determined and found to range between 76.2%–80.3% for the 20% formulation and 64.7%–66.9% for the 40% system. The corresponding average loading for the 20% discs was 3954 ± 79 mg/g and for 40% it was 3293 ± 28 mg/g, as compared to an ideal loading potential of 4000 mg/g and 3000 mg/g, respectively. Table 2 shows the average (n = 3) amount of Theophylline that was entrapped within each disc for each formulation along with the amount of solute (in µg) subsequently released and total % release for each hydrogel formulation.

To determine the mechanism of Theophylline release from the hydrogel discs, the Higuchi, Zero-order and First-order models and the Korsmeyer–Peppas model of solute release were applied. However, as the Higuchi, Zero-order and First-order models all fail to adequately account for release due to swelling or erosion of the polymer matrix [30,31], these were deemed to be unsuitable to describe the mechanism during the initial burst release period. When applied to the period after burst release (i.e., after the 1 h time point), as shown in Table 3, the R² values fluctuated from 0.576–0.926, 0.579–0.927 and 0.772–0.991 for Higuchi, Zero-order and First-order models, respectively. As Korsmeyer–Peppas mathematical modelling considers the effects of multiple mechanisms within a cross-linked polymer system, i.e., a combination of diffusion and erosion, this method was applied across all periods of release from the hydrogels [32,33]. As shown in Table 3, R² values of 0.876–0.985 were determined for the entire duration of drug release. The R² range of 0.964–0.998 for the period after burst release (again after the 1 h time point) compare favorably with the output for the Higuchi zero-order and first-order models. Individual graphical plots for the Zero-order, First-order and Higuchi release profile fits are provided as Supplementary Data (Figures S1 to S16).

### 3.3. Permeability of Rhodamine B through Hydrogel Membranes

**Figure 3:**
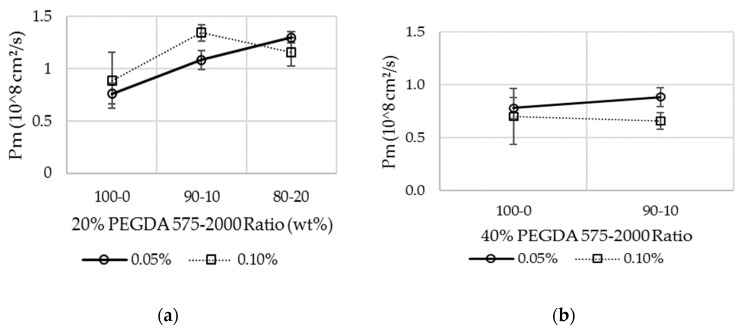
Permeation coefficients of (**a**) 20% and (**b**) 40% PEGDA formulations.

Figure 3 provides the permeation coefficients for each formulation taking account of individual sample thicknesses. Calibration solutions were created by serial dilution of a 0.01% weight/volume Rhodamine B aqueous stock solution and measurement of the fluorescence spectroscopy excitation and emission values at wavelengths of 530 nm and 632 nm, respectively. These data were then used to obtain a suitable calibration curve. Fluorescence measurements taken directly from the hydrogel membrane samples were used to determine the Rhodamine B concentration via simple Beer–Lambert law calculations. It should be noted that data is not presented here for 20% 70-30 and the 40% 70-30 and 80-20 hydrogel discs as they could not be handled without tearing. As such, the data for the 40% hydrogels is presented solely for completeness and is not used for any subsequent considerations of diffusion mechanisms.

Values for the permeability for each of the hydrogel samples tested were achieved by importing the data shown in Figure 3a,b into Equation (4). Whilst the Rhodamine B permeability increased with the increased addition of PEGDA2000 to PEGDA575 for hydrogel discs created at both photoinitiator concentrations from the 20% solution, the permeability for the 80-20 formulation with a 0.1% photoinitiator concentration was seen to decrease as compared to the value for the equivalent 90-10 formulation with 0.1% photoinitiator.

### 3.4. Partition and Diffusion Coefficient Measurements

**Figure 4:**
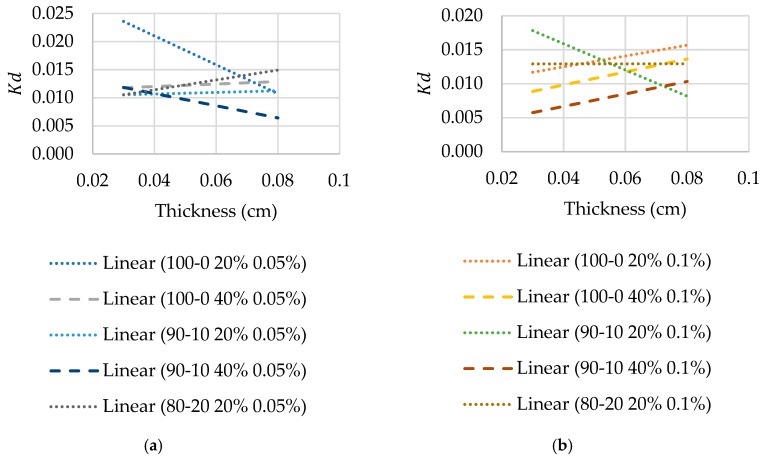
(**a**,**b**) Partition coefficient of each formulation, according to photoinitiator concentration, for thickness range 0.03 to 0.08 cm used to acquire an accurate partition coefficient for each disc used in permeation experiments.

Not unexpectedly, slight differences in thickness of the hydrogel disc used for the permeation experiments affect the value of the diffusion coefficient, *D_m_*, due to the attendant change in the partition coefficient, *K_d_*. Hence, to consider the effect of disc thickness more directly, sets of ~0.3 mm and ~0.8 mm samples (n = 5) were placed into a 3 mL aliquot of the 0.01% weight/volume Rhodamine B solution and left for 3 days. After removal of the discs, the change in concentration of the immersion solution was determined by fluorescence spectroscopy, as before. To account for variations in thickness during permeation of Rhodamine B through the hydrogel discs, a gradient for the plot of *K_d_* against thickness for each formulation was obtained during the permeation test to determine the partition coefficient more accurately, as presented in Figure 4. The diffusion coefficient values were then calculated using Equation (4), with the associated data presented in Table 4. There is an increase in the diffusion coefficient with increasing PEGDA2000 contribution to PEGDA575 in the hydrogel formulation in most cases, with the exception being the 90-10 to 80-20 samples for the 20% polymer samples with 0.1% photoinitiator concentration.

**Figure 5:**
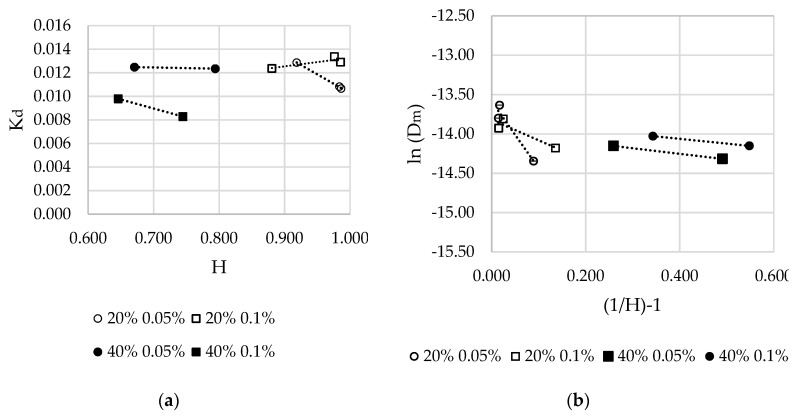
(**a**) Partition coefficients of Rhodamine B in PEGDA 575-2000 formulations according to hydration; (**b**) logarithm of diffusion coefficients of Rhodamine B in PEGDA575-2000 formulations as a function of inverse hydration.

The resulting partition coefficients for the systems of interest, plotted as a function of hydration state (wet weight–dry weight/volume), H, are given in Figure 5a and indicate a state of proportionality between these parameters for all samples studied. To validate the free-volume theory for these hydrogel formulations, plots of diffusion coefficient, *D_m_* against (1/H)-1 as ln(*D_m_*) are provided in Figure 5b. The resulting linear relationship is deemed a positive result, in that it indeed validates the application of the free volume theory, where, typically, the relationship changes with hydration level, as seen to be the case here between the 20% and 40% total polymer concentrations. It is interesting to note that the values for mesh size measured through swelling measurements and model solute diffusion vary slightly for 100-0 hydrogels, especially in the case of those created with the 20% solutions. It is suggested that this is due to the capability of the mixed-molecular-weight hydrogels to expand further and absorb additional water post-polymerisation. While the discs used in swelling studies were allowed to soak to remove any unreacted material for a period of time, those used in release and permeation studies could not be permitted this soaking period. While prepared with 80% water, in the pre-polymerized state, once the discs have been soaked in water for 24 h, the content ranges from 82.1% to 88.3% for those created from the 20% formulations.

### 3.5. Effective Mesh Size

Using a value 0.69 nm as the solute radius for Rhodamine B [34] and the diffusion coefficient values measured experimentally, as reported in Table 4, estimates for effective hydrogel mesh size in the 20% and 40% 100-0, 90-10 and 80-20 formulations created with both photoinitiator concentrations are given in Table 5.

### 3.6. BET-NLDFT Pore Radius Analysis

BET-NLDFT-derived pore radius data for the various PEG575-2000 hydrogels created by crosslinking of 20% and 40% solutions with the 0.05% photoinitiator concentration compared to the estimated mesh size (Å) values^10^ are shown in Table 6. The change in calculated pore radius values that occur as the amount of PEG2000 increases are consistent with the values obtained via the effective mesh size calculations (Table 5). The pore radius of the 90-10 system is significantly higher than that of the 100-0 hydrogel for both the 20% and 40% solutions. A slightly lower than expected pore radius value for the 20% 80-20 PEGDA575-2000 hydrogel is obtained, which is deemed to be due to the fact that the samples studied were specifically designed and fabricated to enable direct solute (liquid) diffusion and release experiments such that there is some variation in the effective sample-to-sample analysis volume herein.

## 4. Discussion

Previous studies have tended to focus on how various molecular weight blends of PEGDA affect the mechanical properties and cell viability of the resulting hydrogels in attempts to mimic bodily tissue such as cartilage [7,35]. As indicated previously, the intended use of the hydrogel systems being developed here is a component of a hybrid tissue engineering scaffold wherein their proposed role is primarily the delivery of pre-loaded nutrient to cells. As such, inherent mechanical properties are not as important as these will be facilitated by other more robust components of the scaffold system. Hence, the choice of augmentation of PEGDA 575 DA with PEGDA 2000 as investigated here reflects the need for hydrogels that offer a higher degree of nutrient diffusion followed by release that extends beyond an initial burst phase.

Although the 70–30 formulation at 20% polymer concentration polymerizes within the (~30 min) time period used here, the Q value was found to decrease compared to that measured for the 80-20 hydrogel but is still greater than that for the 100-0 (PEGDA575) system. This suggests that at both photoinitiator concentrations, the cross-linking efficiency for this 70-30 PEGDA575-2000 20% solution is reduced by the presence of more of the PEGDA2000 polymer chains. By comparison, the trend in the 40% polymer solutions increases with PEGDA2000 contribution but with Q values less than those for the 20% case; therefore, this is interpreted as a limitation in the PEGDA2000 loading. As such, the data for the 40% case is presented here solely for completeness and is not used for subsequent considerations of diffusion mechanisms.

Addition of 10% of PEGDA2000 to 90% PEGDA575 to produce the 20% 90-10 PEGDA575-2000 system causes an increase in permeability of the resulting hydrogel (compared to PEGDA575 alone). Increasing the photoinitiator concentration in the same hydrogel polymer system leads to a slightly higher Pm value. However, this increase is not reflected in the 40% system wherein the PEGDA2000 seems to cause a disruptive influence on the PEGDA575 with an increase in photoinitiator concentration not leading to a higher permeability state. BET-NLDFT-derived pore radius data for the 20% and 40% PEG575-2000 solutions created with 0.05% photoinitiator are consistent with the values obtained via the effective mesh size calculations. SEM analysis of the respective hydrogels (Supplementary Data, Figures S17 to S22) clearly indicates that the 40% 90-10 PEGDA575-2000 system produces a much more closely packed granular microstructure at both photoinitiator concentrations compared to that for the equivalent 20% 90-10 PEGDA575-2000 system. This difference in internal microstructure is especially evident in lower-magnification SEM images (Figures S21 and S22), in which the 40% polymer system shows extensive granularity that is not evident in the 20% polymer formulation, thereby causing the changes in the permeability measurements as noted here. It should be noted that data is not presented here for 20% 80-20 and 40% 70-30 and 80-20 ratio hydrogel discs as they could not be handled without tearing.

Mathematical modelling of the 20% hydrogel, using Higuchi, Zero-order and First-order diffusion mechanisms is not able to entirely represent the main mechanism of release after the burst release. It is noted that while the Zero-order model is not dependent on concentration of the solute, the first-order approach does take this into account. The slight linearity in the data, as shown in Table 3 and the associated Supplementary Data (Figures S1 to S16), may indicate that, in some instances, these models are able to account for the observed behaviour. By comparison, the Korsmeyer–Peppas model fits the data well, with a correlation coefficient of 0.876 < R² < 0.985 across all periods of release, increasing to 0.964 < R² < 0.998 after the initial burst release, i.e., after 1 h, indicating that the release mechanism better follows the kinetics associated with this model.

The n exponent of the Korsmeyer–Peppas model indicates the type of diffusion that occurs, with n < 0.45 described as quasi-Fickian diffusion, n = 0.45 as Fickian diffusion, 0.45 n < 0.89 as anomalous or non-Fickian diffusion and n > 0.89 as Case II or non-Fickian Case II transport [36]. The 20% PEGDA575-2000 hydrogel systems created here have values of 0.035 < n < 0.083 and 6.43 < n < 12.46, indicating that a quasi-Fickian mechanism of release is most likely, where the Theophylline is partly diffused through a swollen matrix and the water-filled pores in the matrix [37,38].

## 5. Conclusions

Tight mesh or microporous networks are typically formed when low-molecular-weight polymers are used to create a hydrogel matrix wherein mesh size is largely influenced by the length of the molecular chains available. Incorporation of a higher-molecular-weight polymer at different ratios and concentrations offers an opportunity to control diffusion and the subsequent release of fluids therein by providing areas of increased mesh size within the network while still maintaining a small microporous system overall. Hydrogel matrices created by UV polymerization of various PEGDA575-2000 formulations have been shown to be capable of storing and releasing nutrient solutions at room temperature, in a manner that offers a degree of control that is not available from the PEGDA-575 system alone. Both the amount of the higher-molecular-weight component present and the photoinitiator concentration are seen to be important in this regard. However, handling issues limited the sample sets for analysis to 20% 100-0, 90-10, 80-20 and 40% 100-0 and 90-10.

While there is burst release phenomena within the first hour of release, the systems can retain stored solute and release it more slowly thereafter. In the case of the 20% hydrogels, the Korsmeyer–Peppas model of solute diffusion is found to be the best fit for the permeability data obtained for these mixed-molecular-weight hydrogel systems, while a quasi-Fickian mechanism is the most likely form of the subsequent fluid release. This suggests that the range of 20% PEGDA575-2000 mesh sizes produced in this way can allow for the more prolonged release profile sought for the delivery of nutrients within tissue engineering scaffolds, although the precise values of the diffusion and release characteristics at body temperature need to be further assessed.

## Figures and Tables

**Table 1:** Mesh size calculations for 100-0 PEGDA575-2000 hydrogel at 20% and 40% concentration.

Polymer Concentration	Photoinitiator Concentration	Distance between Crosslinks, Mc (Equation (6))	Estimated mesh size (Å) (Equation (9))
**20%**	0.05%	259.03	19.08
	0.10%	260.56	19.27
**40%**	0.05%	212.77	14.87
	0.10%	225.88	14.46

**Table 2:** Theophylline content (µg), amount released (µg) and total % released from at 20% and 40% PEGDA575-2000 hydrogel discs in 0.8% saline solution after 24 h (n = 3).

Blend Formulation		Irgacure 2959 Concentration	Polymer Concentration	Theophylline Content (µg)	Theophylline Released (µg)	Total % Release
**20%**	100-0	0.05%	19.63	128.6 ± 4.8	21.7 ± 0.5	16.9 ± 0.4
		0.10%	21.33	158.7 ± 4.9	18.4 ± 1.2	11.6 ± 0.8
	90-10	0.05%	23.76	150.1 ± 0.4	21.4 ± 0.7	14.3 ± 0.5
		0.10%	21.90	170.9 ± 3.0	25.6 ± 0.3	15.0 ± 0.2
	80-20	0.05%	21.72	124.6± 11.5	18.0 ± 1.1	14.4 ± 0.9
		0.10%	20.67	139.9 ± 0.5	20.7 ± 0.4	14.8 ± 0.3
	70-30	0.05%	19.21	127.6 ± 6.0	18.1 ± 0.8	14.2 ± 0.7
		0.10%	19.08	127.6 ± 2.4	18.6 ± 0.4	14.6 ± 0.3
**40%**	100-0	0.05%	33.41	124.2 ± 1.2	17.1 ± 1.2	13.8 ± 0.9
		0.10%	34.25	132.0 ± 3.8	14.5 ± 0.6	11.0 ± 0.4
	90-10	0.05%	35.22	101.3 ± 0.4	15.9 ± 0.8	15.7 ± 0.7
		0.10%	33.69	108.2 ± 2.7	18.7 ± 0.6	17.3 ± 0.5
	80-20	0.05%	34.13	104.9 ± 1.3	15.9 ± 0.3	15.2 ± 0.2
		0.10%	34.17	100.2 ± 1.8	16.5 ± 0.1	16.5 ± 0.1
	70-30	0.05%	33.69	109.0 ± 1.7	17.4 ± 0.3	16.0 ± 0.2
		0.10%	34.49	96.6 ± 1.6	16.3 ± 0.5	16.9 ±0.5

**Table 3:** R², n and k values obtained from Korsmeyer–Peppas models and R² values after 1 h from Higuchi, zero- and first-order models.

Blend Formulation		Irgacure 2959 Concentration Concentration	Korsmeyer Model	n	k	Korsmeyer Model 1 h +	Zero Order 1 h +	First Order 1 h +	Higuchi 1 h +
**20%**	100-0	0.05%	0.965	0.055	11.51	0.991	0.739	0.742	0.899
		0.10%	0.976	0.046	8.28	0.984	0.916	0.917	0.988
	90-10	0.05%	0.914	0.070	8.90	0.985	0.667	0.670	0.848
		0.10%	0.924	0.075	9.01	0.964	0.576	0.579	0.772
	80-20	0.05%	0.910	0.051	10.12	0.980	0.857	0.858	0.959
		0.10%	0.876	0.058	9.95	0.986	0.820	0.821	0.941
	70-30	0.05%	0.880	0.042	10.62	0.975	0.926	0.927	0.991
		0.10%	0.942	0.040	11.04	0.998	0.793	0.795	0.935
**40%**	100-0	0.05%	0.940	0.055	8.99	0.982	0.676	0.676	0.850
		0.10%	0.985	0.046	6.43	0.992	0.676	0.676	0.900
	90-10	0.05%	0.953	0.083	8.93	0.980	0.651	0.655	0.835
		0.10%	0.910	0.077	10.32	0.976	0.621	0.624	0.812
	80-20	0.05%	0.876	0.079	8.86	0.991	0.788	0.790	0.926
		0.10%	0.893	0.066	10.45	0.988	0.810	0.813	0.937
	70-30	0.05%	0.925	0.035	12.47	0.986	0.905	0.906	0.988
		0.10%	0.942	0.045	12.26	0.989	0.856	0.857	0.963

**Table 4:** Partition coefficient and diffusion coefficient for Rhodamine B on various PEGDA575-2000 blend formulations (n = 3).

Blend Formulation		Irgacure 2959 Concentration		Kd (×10^2^) (mean ± SD)		Dm (×10^−8^ cm²/s) (mean ± SD)	
**20%**	100-0	**0.05%**		1.29	± 0.019	58.91	± 3.49
	90-10			1.07	± 0.002	101.81	± 7.88
	80-20			1.08	± 0.005	119.96	± 6.60
	100-0	**0.10%**		1.24	±0.016	71.28	± 18.61
	90-10			1.34	±0.014	100.51	± 2.58
	80-20			1.29	±0.000	89.90	± 10.28
**40%**	100-0	**0.05%**		1.25	±0.003	62.48	± 18.41
	90-10			1.23	±0.005	71.61	± 7.78
	100-0	**0.10%**		0.98	±0.009	72.08	± 11.53
	90-10			0.83	±0.051	85.27	± 31.47

**Table 5:** Estimated mesh sizes based on the Ratner and Miller equation for determination mesh size in microporous polymers [29].

Polymer Concentration	Irgacure 2959 Concentration	Formulation	Effective Mesh Size (Å) (Equation (10))
**20%**	**0.05%**	**100-0**	13.9
		**90-10**	17.3
		**80-20**	18.8
	**0.10%**	**100-0**	14.6
		**90-10**	17.2
		**80-20**	16.3
**40%**	**0.05%**	**100-0**	13.9
		**90-10**	14.9
	**0.10%**	**100-0**	14.8
		**90-10**	17.3

**Table 6:** BET-NLDFT-derived pore radius data (Å) for PEG575-2000 hydrogels created by crosslinking of 20% and 40% solutions with the 0.05% photoinitiator concentration compared to the estimated mesh size (Å).

Polymer Concentration	Formulation	BET- NLDFT Pore Radius (Å)	Effective Mesh Size (Å) (Equation (10))
**20%**	**100-0**	13.85	13.9
	**90-10**	15.85	17.3
	**80-20**	14.48	18.8
**40%**	**100-0**	11.91	13.9
	**90-10**	13.85	14.9

## Supplementary Materials

The following are available online at https://www.mdpi.com/1996-1944/12/20/3381/s1.
