# Supplementary material for: Controlling Fluid Diffusion and Release through Mixed-Molecular-Weight Poly(ethylene) Glycol Diacrylate (PEGDA) Hydrogels
Source: Materials (Basel). 2019 Oct 16;12(20):3381. doi: 10.3390/ma12203381 (PMC6829336; doi:10.3390/ma12203381)
Supplement: Supplementary file 1 [file materials-12-03381-s001.pdf]

materials

Supplementary

Controlling Fluid Diffusion and Release through Mixed-Molecular-Weight Poly(ethylene) Glycol Diacrylate (PEGDA) Hydrogels

Kieran O'Donnell, Adrian Boyd and Brian J. Meenan *

Nanotechnology and Integrated BioEngineering Centre (NIBEC), School of Engineering, Ulster University, Shore Road, Newtownabbey BT37 0QB, UK; ODonnell-k9@ulster.ac.uk (K.O.); ar.boyd@ulster.ac.uk (A.B.)
* Correspondence to: bj.meenan@ulster.ac.uk; Tel.: +44-28-90368939

Received: 12 September 2019; Accepted: 8 October 2019; Published: 16 October 2019

Plots of zero-order, first-order and Higuchi fluid (solute) release profile fits for PEGDA575-2000 hydrogel formulations after the 1 hour based on the data sets presented in Table 3.

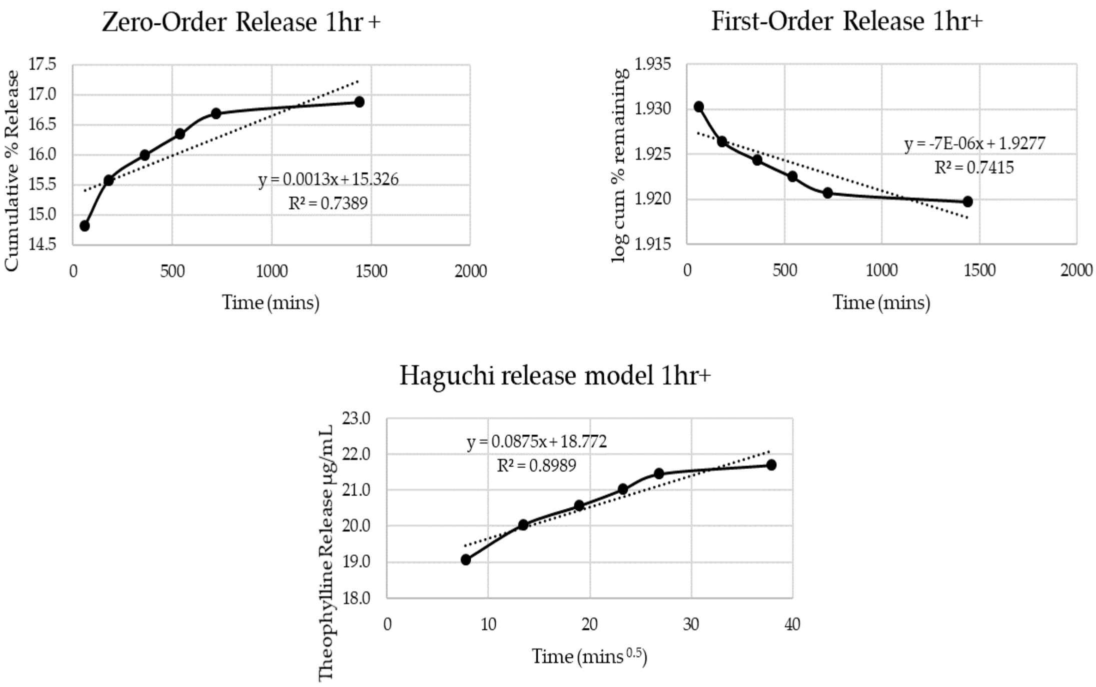

Figure S1. Zero-order, First-order and Higuchi release profiles for 20% PEGDA575-2000 100–0 at 0.05% photoinitiator concentration.

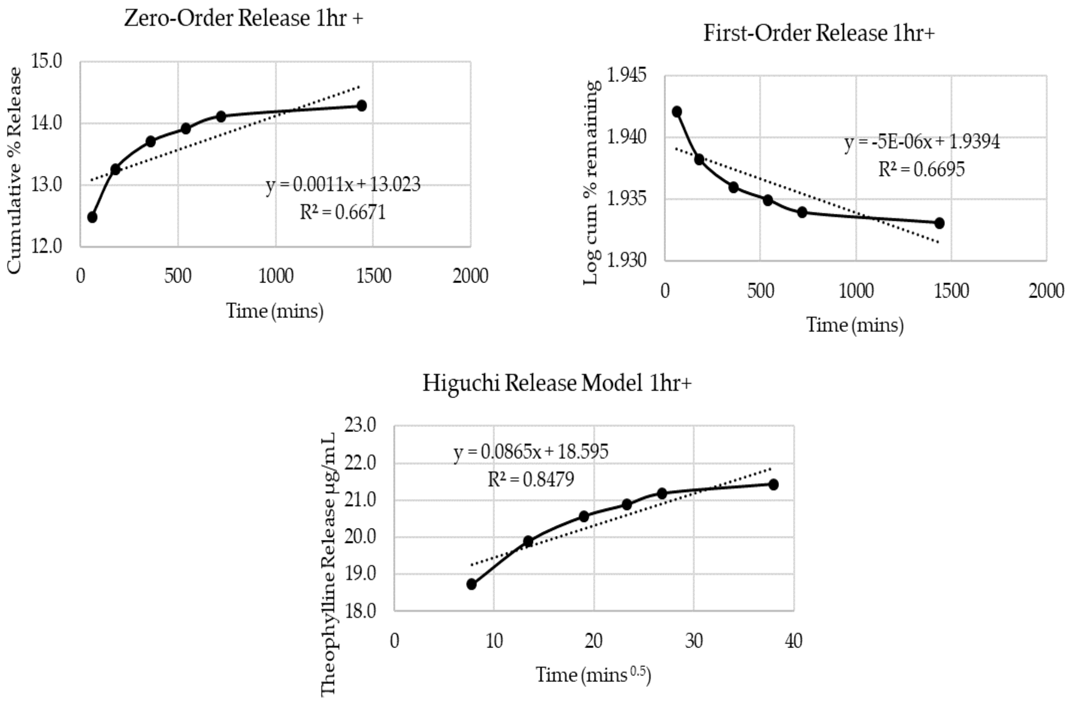

Figure S2. Zero-order, First-order and Higuchi release profiles for 20% PEGDA575-2000 90–10 at 0.05% photoinitiator concentration.

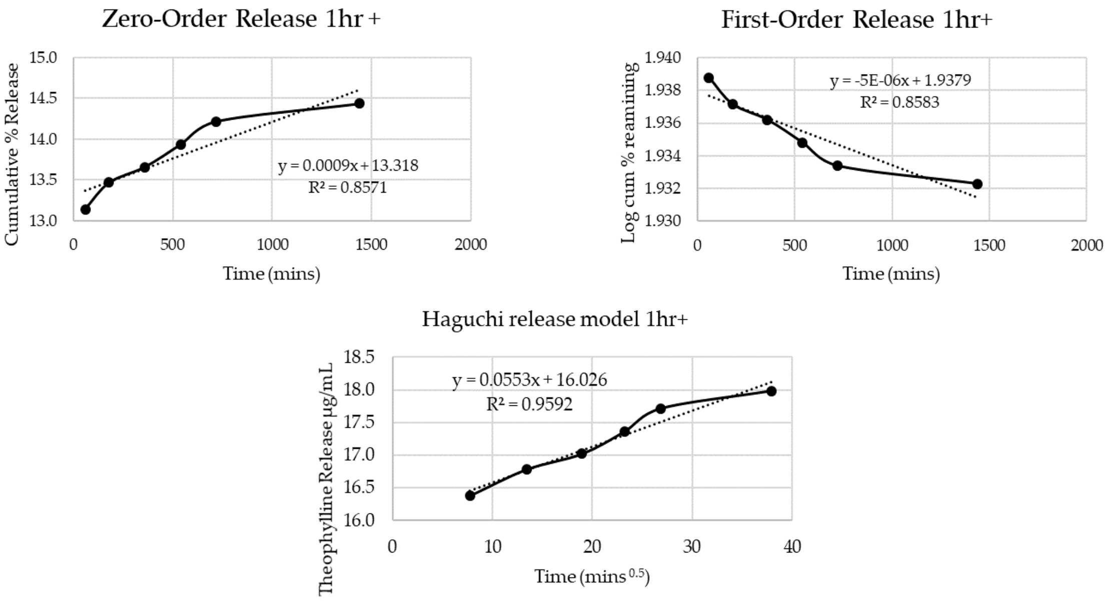

Figure S3. Zero-order, First-order and Higuchi release profiles for 20% PEGDA575-2000 80–20 at 0.05% photoinitiator concentration.

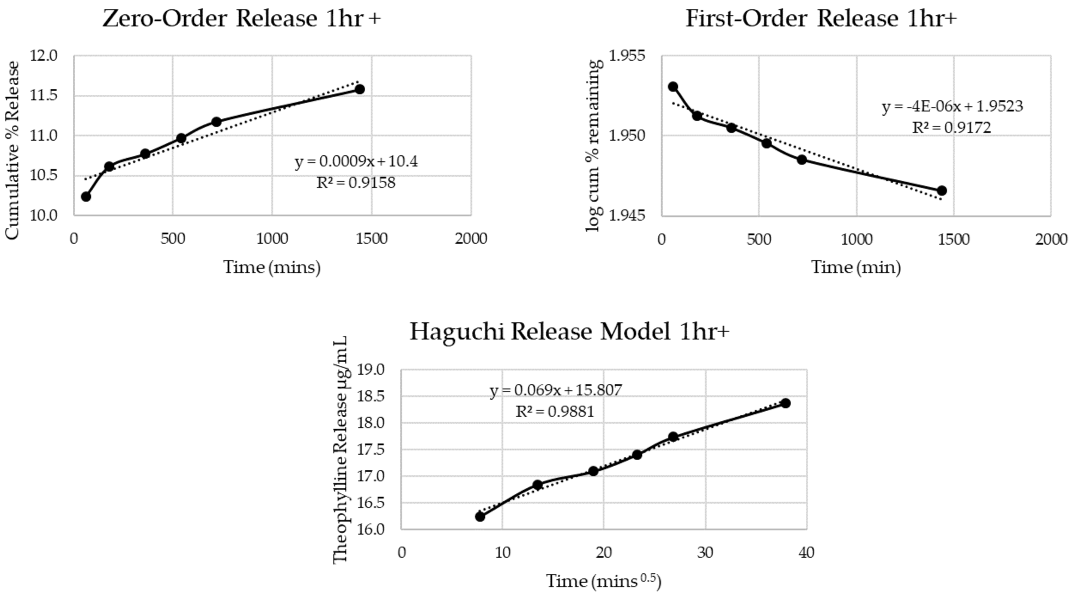

Figure S4. Zero-order, First-order and Higuchi release profiles for 20% PEGDA575-2000 70–30 at 0.05% photoinitiator concentration.

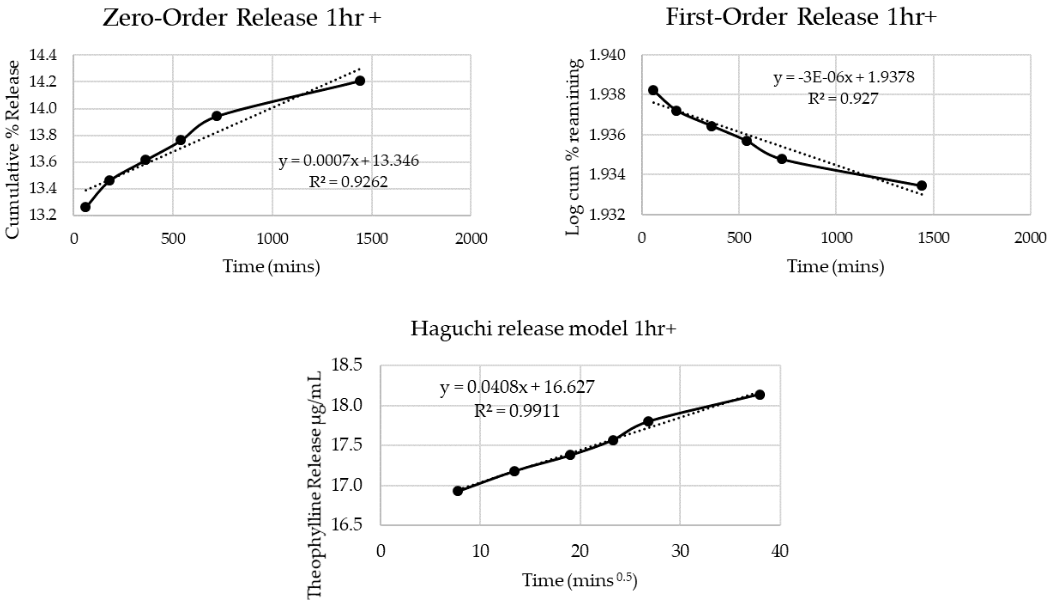

Figure S5. Zero-order, First-order and Higuchi release profiles for 20% PEGDA575-2000 100–0 at 0.1% photoinitiator concentration.

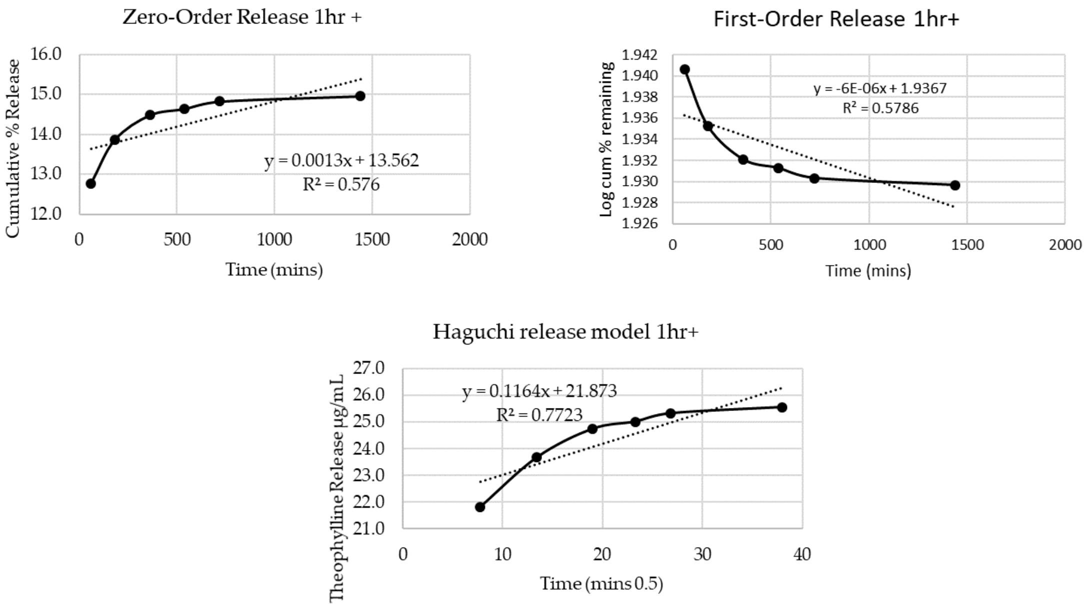

Figure S6. Zero-order, First-order and Higuchi release profiles for 20% PEGDA575-2000 90–10 at 0.1% photoinitiator concentration.

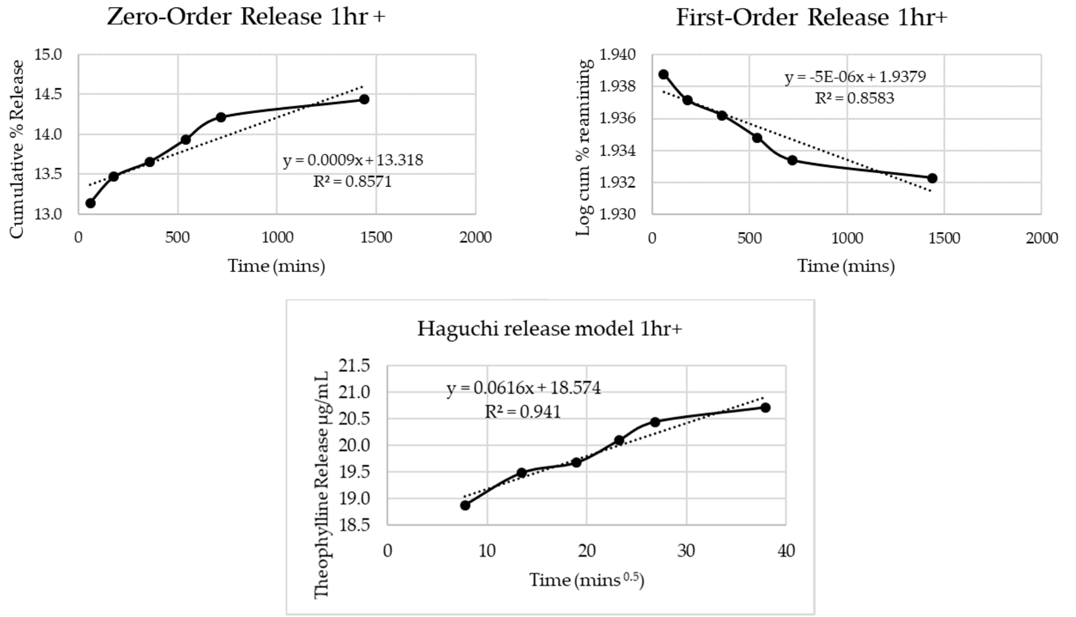

Figure S7. Zero-order, First-order and Higuchi release profiles for 20% PEGDA575-2000 80–20 at 0.1% photoinitiator concentration.

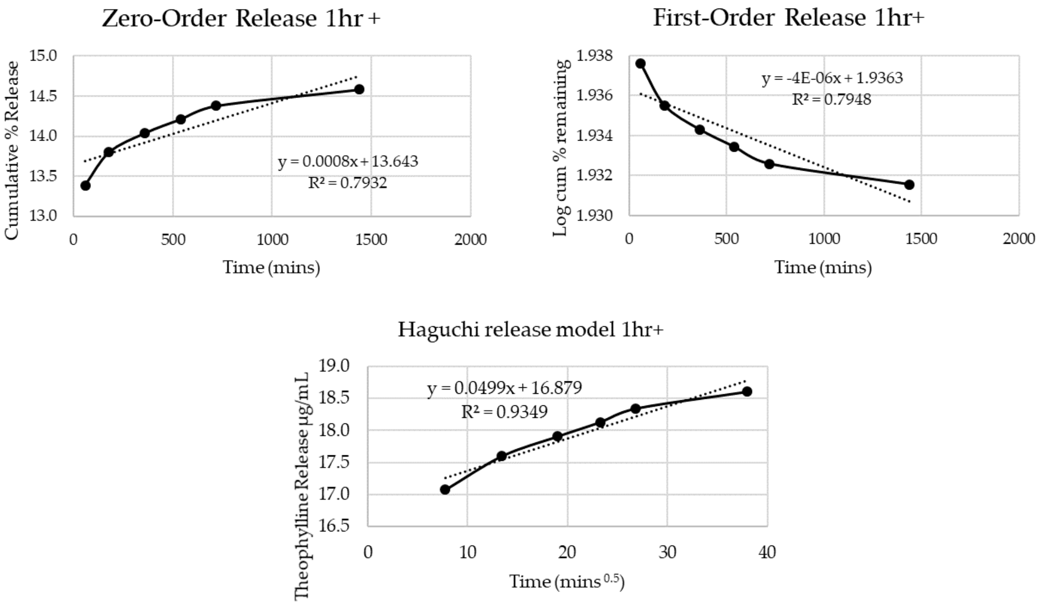

Figure S8. Zero-order, First-order and Higuchi release profiles for 20% PEGDA575-2000 70–30 at 0.1% photoinitiator concentration.

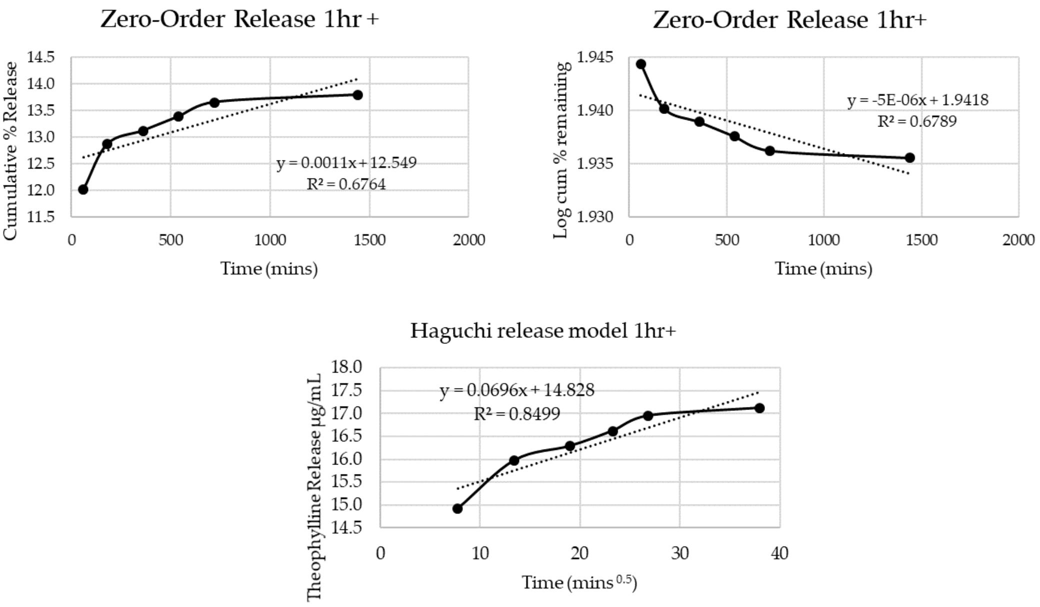

Figure S9. Zero-order, First-order and Higuchi release profiles for 40% PEGDA575-2000 100–0 at 0.05% photoinitiator concentration.

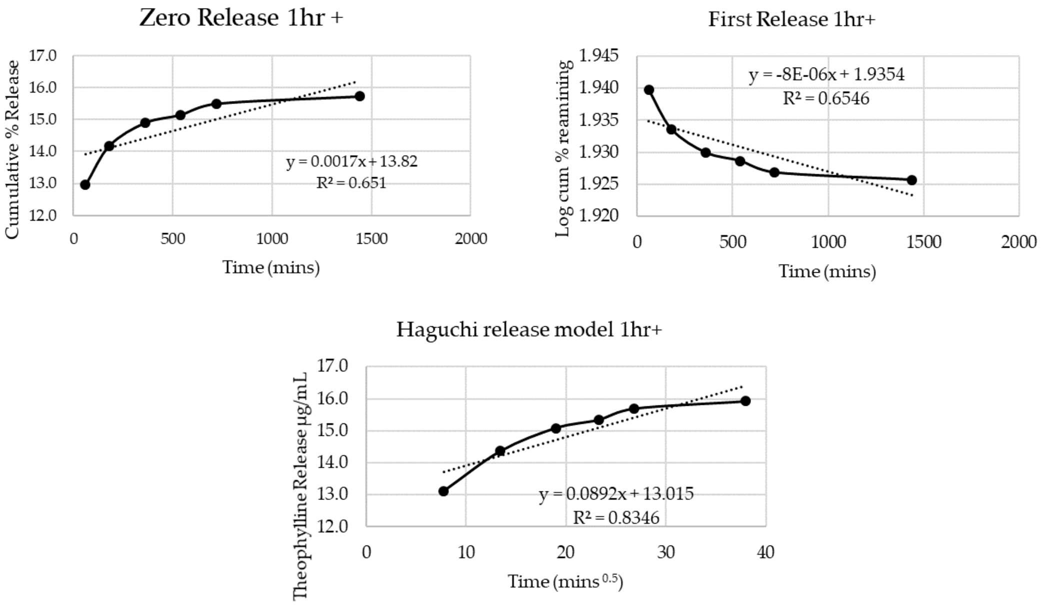

Figure S10. Zero-order, First-order and Higuchi release profiles for 40% PEGDA575-2000 90–10 at 0.05% photoinitiator concentration.

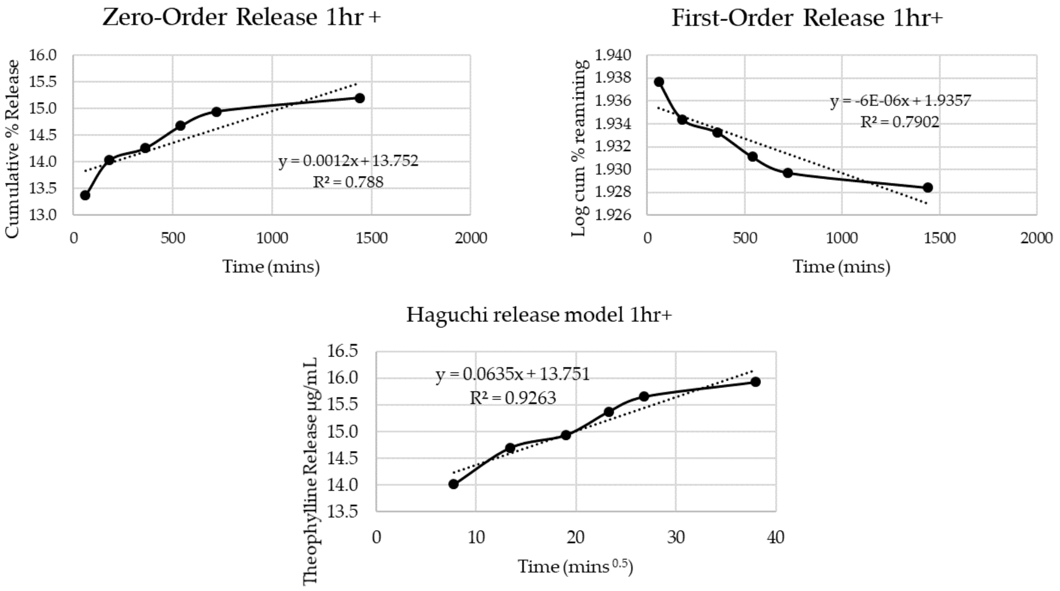

Figure S11. Zero-order, First-order and Higuchi release profiles for 40% PEGDA575-2000 80–20 at 0.05% photoinitiator concentration.

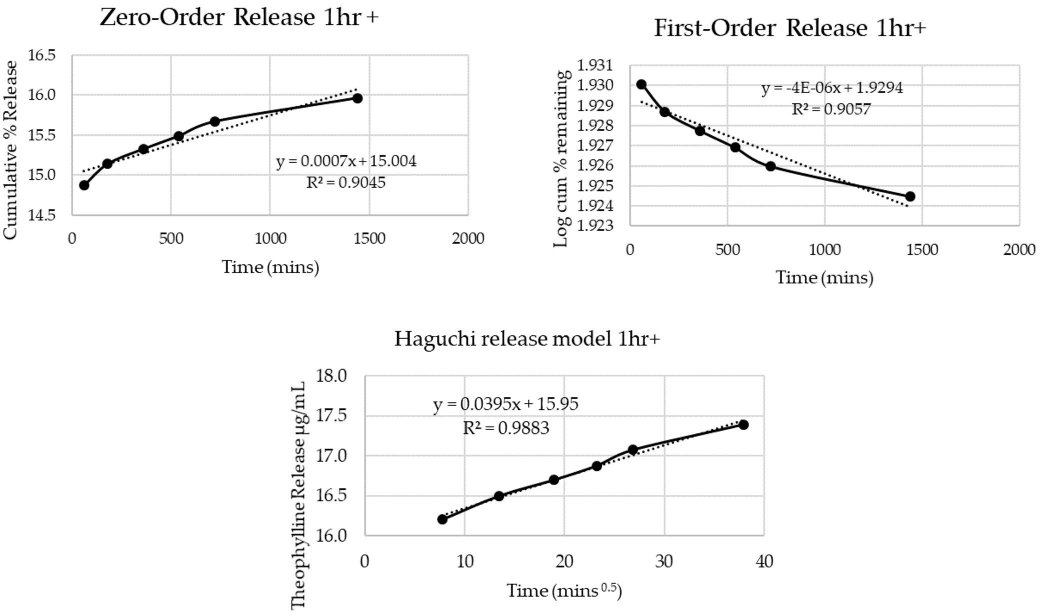

Figure S12. Zero-order, First-order and Higuchi release profiles for 40% PEGDA575-2000 70–30 at 0.05% photoinitiator concentration.

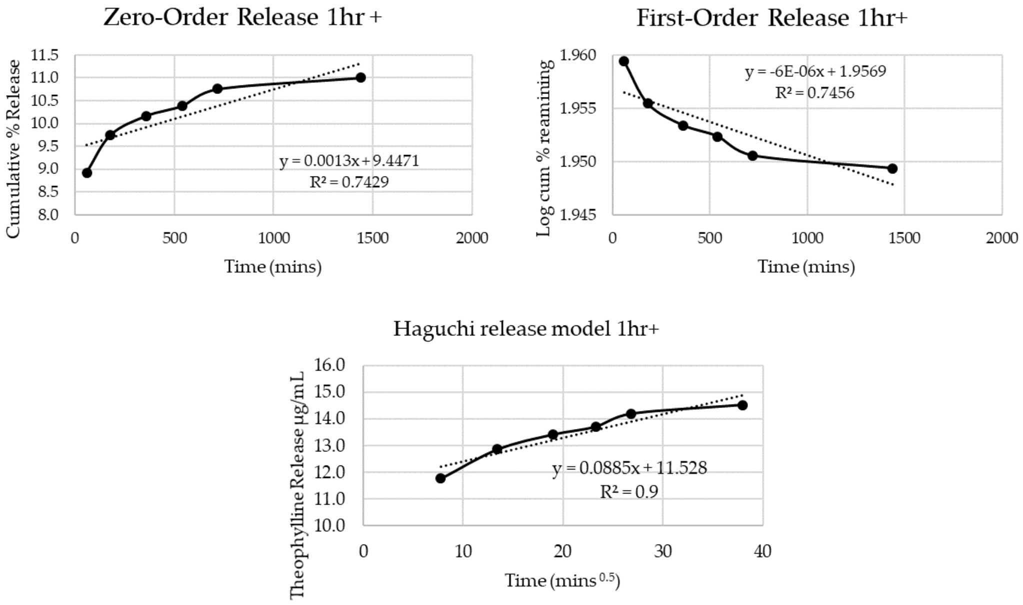

Figure S13. Zero-order, First-order and Higuchi release profiles for 40% PEGDA575-2000 100–0 at 0.1% photoinitiator concentration.

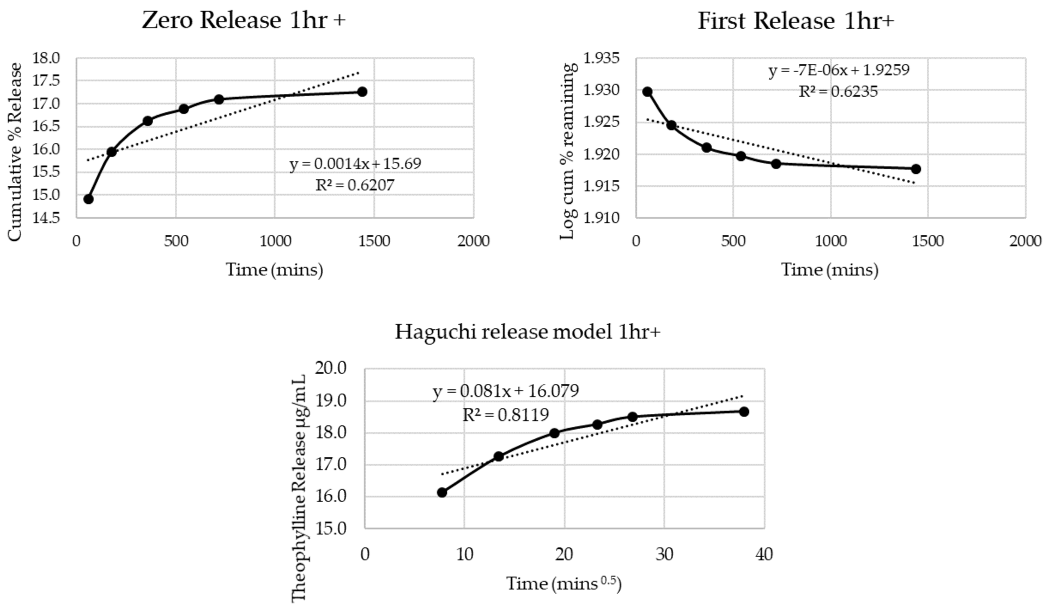

Figure S14. Zero-order, First-order and Higuchi release profiles for 40% PEGDA575-2000 90–10 at 0.1% photoinitiator concentration.

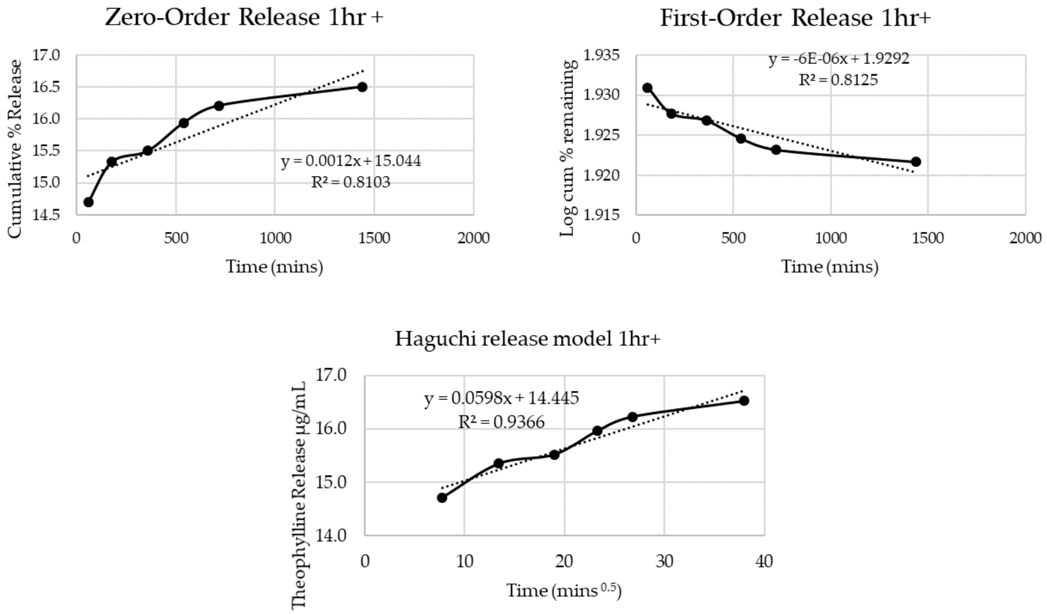

Figure S15. Zero-order, First-order and Higuchi release profiles for 40% PEGDA575-2000 80–20 at 0.1% photoinitiator concentration.

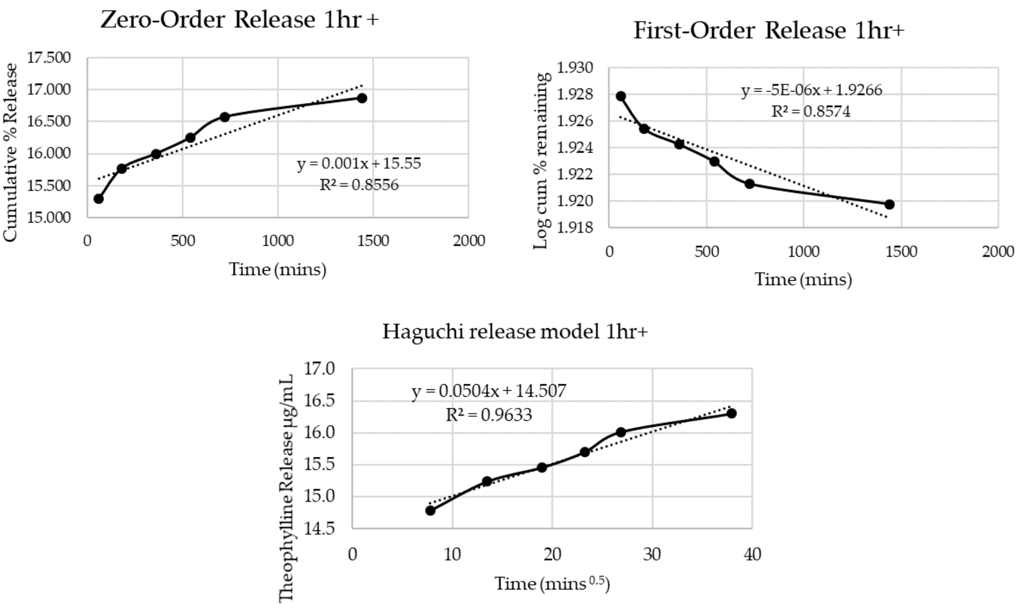

Figure S16. Zero-order, First-order and Higuchi release profiles for 40% PEGDA575-2000 70–30 at 0.1% photoinitiator concentration.

Scanning Electron Microscopy (SEM) Analysis of PEGDA575-2000 hydrogel formulations recorded on a Jeol JSM-6010PLUS/LV instrument operating at an electron beam voltage of 10kV for samples that had been thoroughly dried in vacuum and gold coated before imaging.

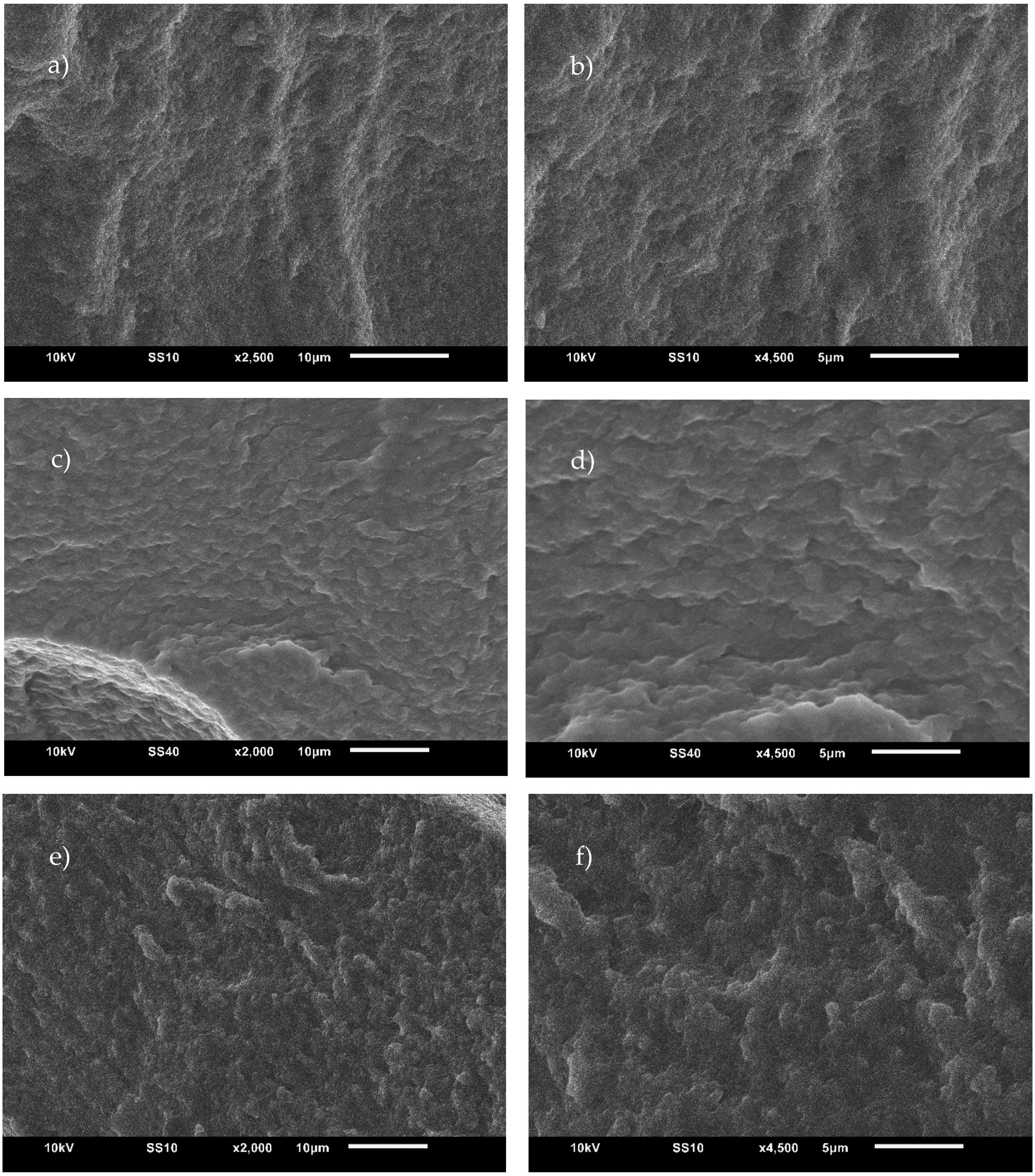

Figure S17. 20% PEGDA575-2000 hydrogel samples at 05% photoinitiator concentration, (**a**,**b**) 100–0; (**c**,**d**) 90–10; (**e**,**f**) 80–20.

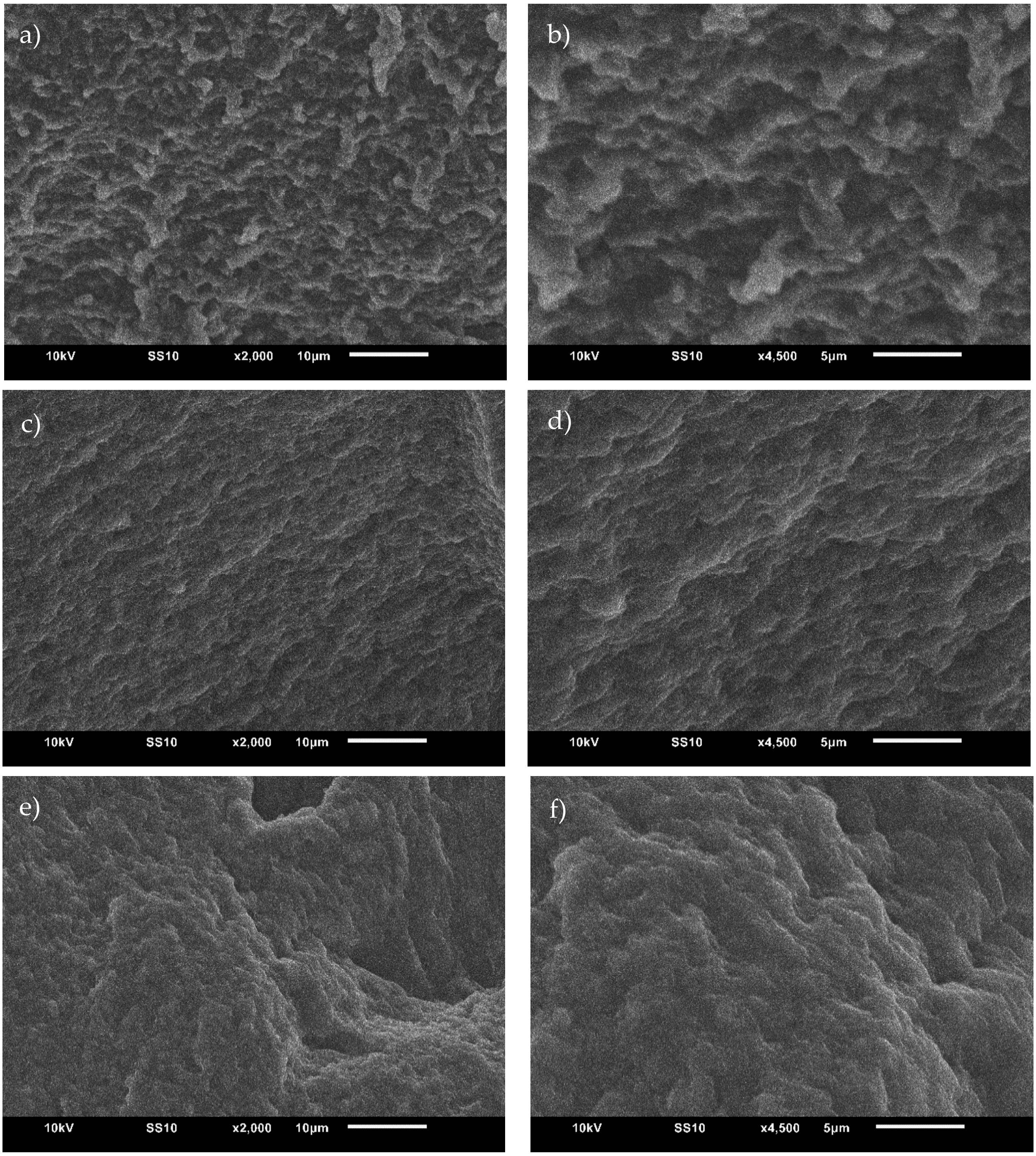

Figure S18. 20% PEGDA575-2000 hydrogel samples at 0.1% photoinitiator concentration, (**a**,**b**) 100–0; (**c**,**d**) 90-10; (**e**,**f**) 80–20.

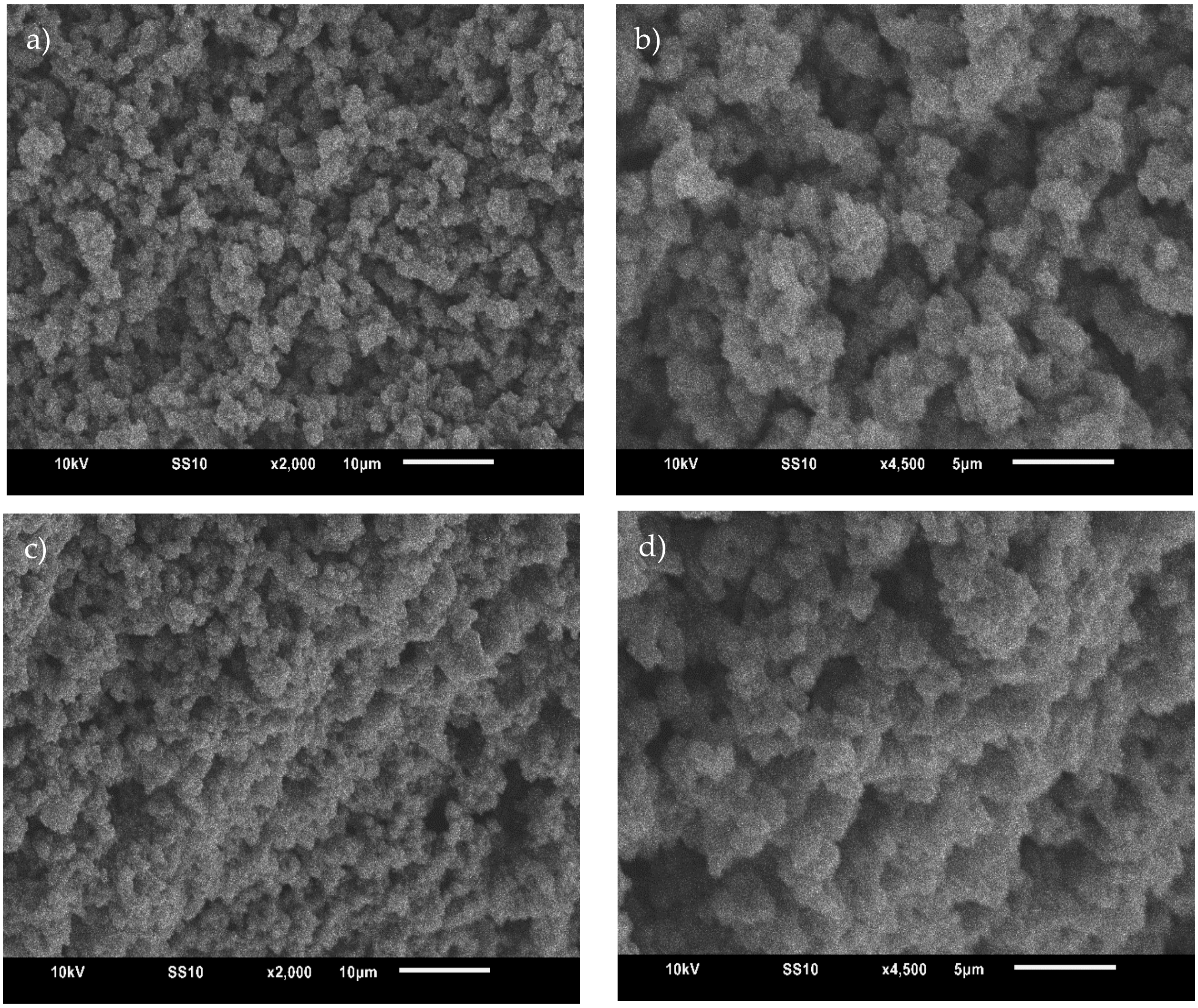

Figure S19. 40% PEGDA575-2000 hydrogel samples at 0.05% photoinitiator concentration, (**a**,**b**) 100–0; (**c**,**d**) 90–10.

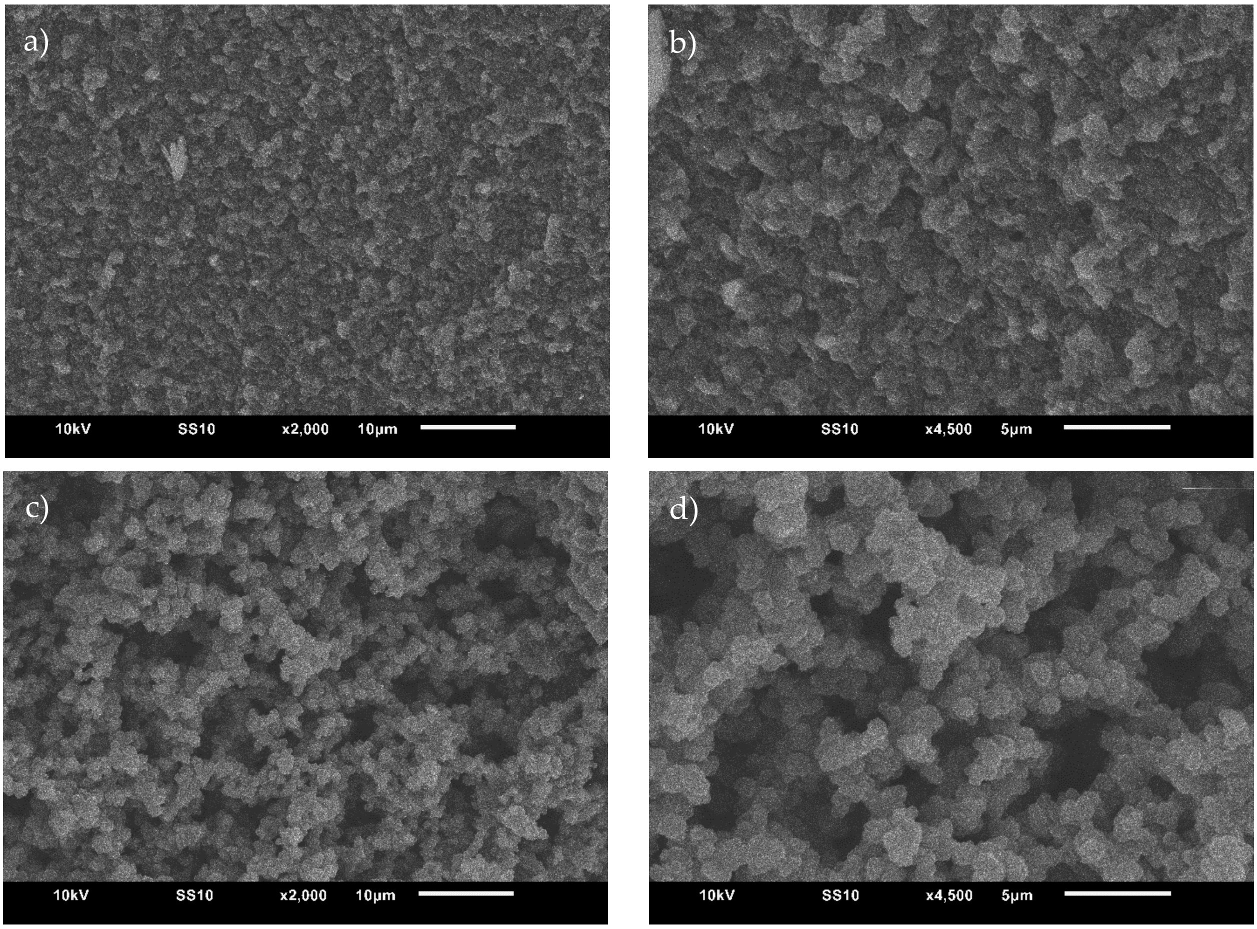

Figure S20. 40% PEGDA575-2000 hydrogel samples at 0.1% photoinitiator concentration, (**a**,**b**) 100–0; (**c**,**d**) 90–10.

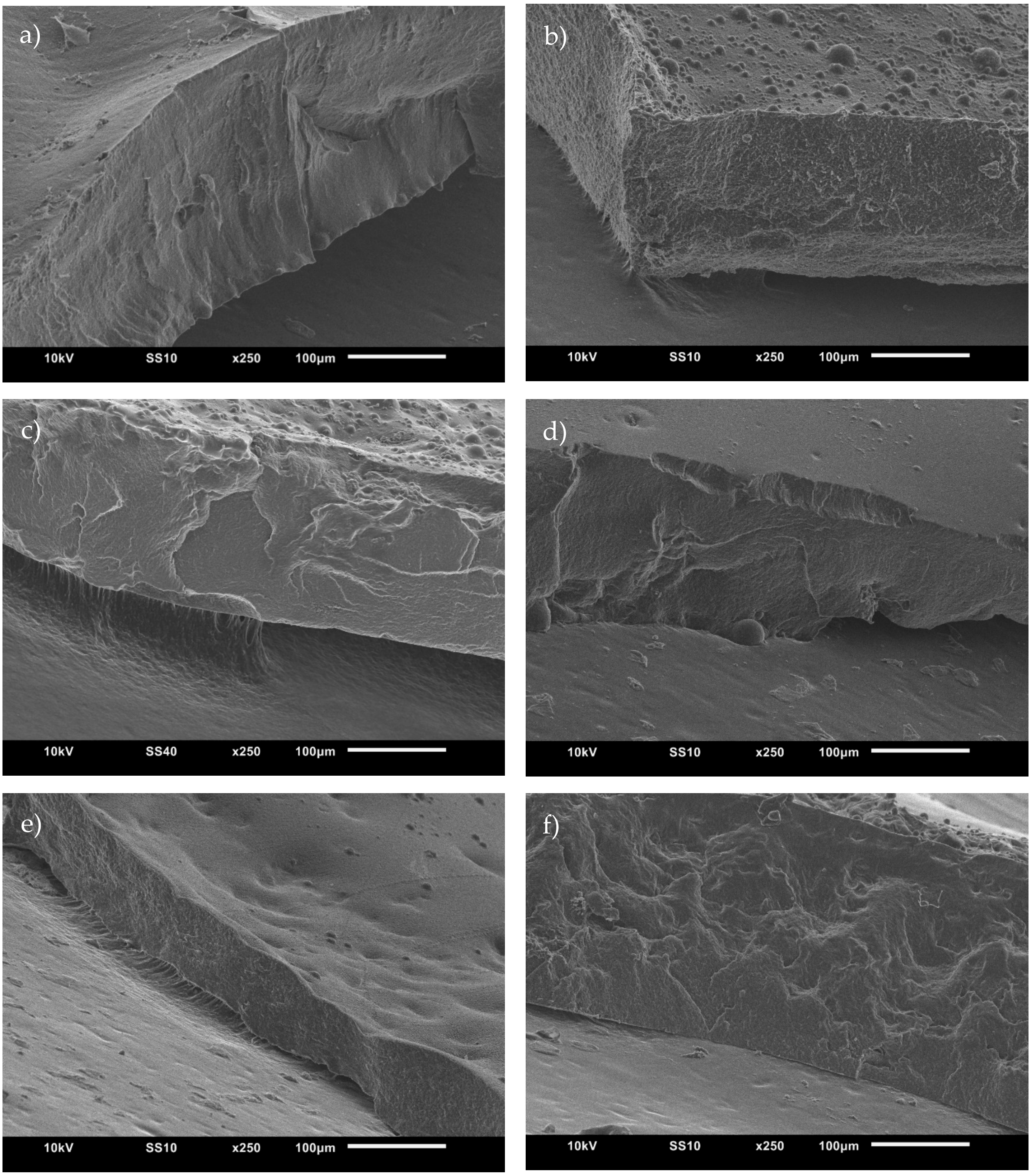

Figure S21. 20% PEGDA575-2000 hydrogels created with a 0.05% photoinitiator concentration: (**a**) 100–0; (**c**) 90–10; (**e**) 80–20; 0.1% photoinitiator concentration (**b**) 100–0; (**d**) 90–10; (**f**) 80–20.

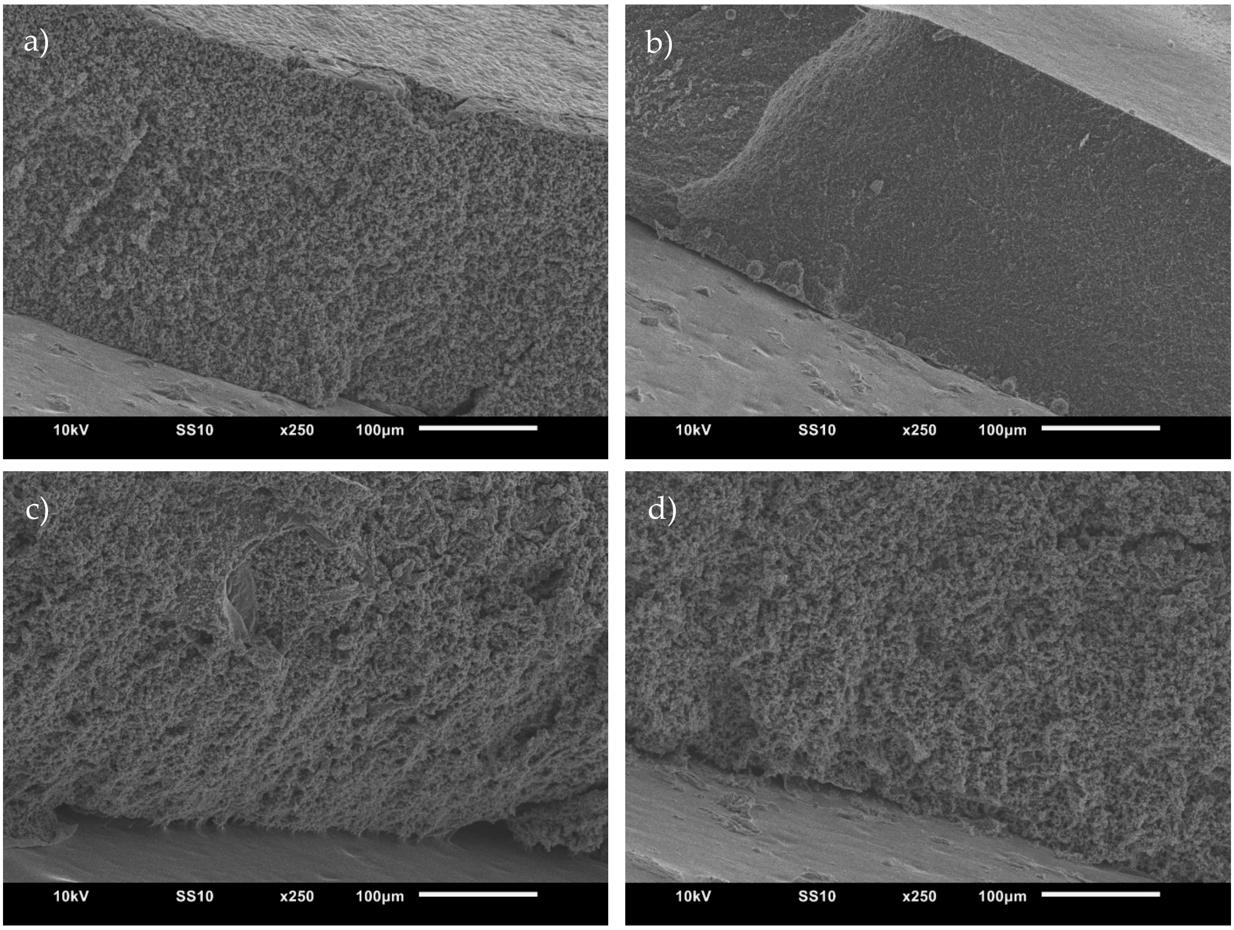

Figure S22. 40% PEGDA575-2000 hydrogels created with 0.05% Photoinitiator concentration: (**a**) 100–0; (**c**) 90–10; 0.1% photoinitiator concentration: (**b**) 100–0; (**d**) 90–10.
